# Fluoroquinolone Resistance Mechanisms of *Shigella flexneri* Isolated in Bangladesh

**DOI:** 10.1371/journal.pone.0102533

**Published:** 2014-07-16

**Authors:** Ishrat J. Azmi, Bijay K. Khajanchi, Fatema Akter, Trisheeta N. Hasan, Mohammad Shahnaij, Mahmuda Akter, Atanu Banik, Halima Sultana, Mohammad A. Hossain, Mohammad K. Ahmed, Shah M. Faruque, Kaisar A. Talukder

**Affiliations:** 1 Centre for Food and Water Borne Diseases, International Centre for Diarrhoeal Disease Research, Bangladesh, Dhaka, Bangladesh; 2 Drug Testing Laboratory, Institute of Public Health, Dhaka, Bangladesh; Baylor College of Medicine, United States of America

## Abstract

**Objective:**

To investigate the prevalence and mechanisms of fluoroquinolone resistance in *Shigella* species isolated in Bangladesh and to compare with similar strains isolated in China.

**Methods:**

A total of 3789 *Shigella* isolates collected from Clinical Microbiology Laboratory of icddr,b, during 2004–2010 were analyzed for antibiotic susceptibility. Analysis of plasmids, plasmid-mediated quinolone-resistance genes, PFGE, and sequencing of genes of the quinolone-resistance-determining regions (QRDR) were conducted in representative strains isolated in Bangladesh and compared with strains isolated in Zhengding, China. In addition, the role of efflux-pump was studied by using the efflux-pump inhibitor carbonyl cyanide-m-chlorophenylhydrazone (CCCP).

**Results:**

Resistance to ciprofloxacin in *Shigella* species increased from 0% in 2004 to 44% in 2010 and *S. flexneri* was the predominant species. Of *Shigella* spp, ciprofloxacin resistant (Cip^R^) strains were mostly found among *S. flexneri* (8.3%), followed by *S. sonnei* (1.5%). Within *S. flexneri* (n = 2181), 14.5% were resistance to ciprofloxacin of which serotype 2a was predominant (96%). MIC of ciprofloxacin, norfloxacin, and ofloxacin were 6–32 mg/L, 8–32 mg/L, and 8–24 mg/L, respectively in *S. flexneri* 2a isolates. Sequencing of QRDR genes of resistant isolates showed double mutations in *gyr*A gene (Ser^83^Leu, Asp^87^Asn/Gly) and single mutation in *par*C gene (Ser^80^Ile). A difference in amino acid substitution at position 87 was found between strains isolated in Bangladesh (Asp^87^Asn) and China (Asp^87^Gly) except for one. A novel mutation at position 211 (His→Tyr) in *gyr*A gene was detected only in the Bangladeshi strains. Susceptibility to ciprofloxacin was increased by the presence of CCCP indicating the involvement of energy dependent active efflux pumps. A single PFGE type was found in isolates from Bangladesh and China suggesting their genetic relatedness.

**Conclusions:**

Emergence of fluoroquinolone resistance in *Shigella* undermines a major challenge in current treatment strategies which needs to be followed up by using empirical therapeutic strategies.

## Introduction

Shigellosis caused by *Shigella* species is endemic throughout the world, and is one of the most important causes of global childhood mortality and morbidity. Globally, every year there are about 165 million cases of *Shigella* infection and 1.1 million *Shigella*-related deaths [1]. *Shigella* is transmitted efficiently in low-dose via fecal-oral route in areas, with poor hygienic conditions, and limited access to clean and potable water [2]. Of four *Shigella* species, shigellosis is predominantly caused by *S. flexneri* in the developing world especially in Asia, whereas *S. sonnei* is the predominant causative agent of this disease in developed as well as industrialized countries [1]. A recent multicenter study of the epidemiology and microbiology of shigellosis in Asia revealed that the incidence of this disease might even exceed previous estimations, as *Shigella* DNA could also be detected in up to one-third of culture-negative specimens [3].

Antimicrobial therapy is effective for the treatment of shigellosis. Increased resistance to commonly used antibiotics including ampicillin, streptomycin, sulfamethoxazole-trimethoprim, nalidixic acid and tetracycline among *Shigella* poses a major therapeutic challenge to control this disease [4]. One of the reasons for emergence of multi-drug resistant *Shigella* spp. is the unique capability of the pathogen to acquire resistance factors (transmissible genes) from the environment or from other bacteria [5]. Besides, indiscriminate use of antibiotics for the treatment of human infection and in animal husbandries in endemic areas triggers the increase of resistance to newer antibiotics [6]. Ciprofloxacin, a third generation fluoroquinolone, has been used successfully for the treatment of infectious diseases including shigellosis. Following a successful clinical trial, ciprofloxacin has been recommended for the treatment of both childhood and adult shigellosis caused by multiple antibiotic resistant *Shigella* spp. in Bangladesh [7]. However, this antibiotic is no longer effective for the treatment of shigellosis in south Asian region including Bangladesh because of the emergence of fluoroquinolone resistant *S*. *dysenteriae* type 1 and their (same clone) dissemination across the countries [8], [9].

Quinolone resistance emerges due to i) point mutations that result in amino acid substitution in chromosomal genes for DNA gyrase and topoisomerase IV, the targets of quinolone action and ii) changes in expression of efflux pumps and outer membrane permeability that control the accumulation of these agents inside the bacterial cell [10], [11]. In addition, a novel mechanism of plasmid-mediated quinolone resistance has recently been reported, and this involves DNA gyrase protection by a protein from the pentapeptide repeat family called Qnr [12], [13]. In Gram-negative organisms, the primary target of fluoroquinolones is the enzyme DNA gyrase, which is essential for DNA synthesis [14]. DNA gyrase consists of two A and two B subunits, encoded by the *gyr*A and *gyr*B genes, respectively. Most mutations have been shown to reside in a small region near the start of the *gyr*A gene, termed as quinolone resistance-determining region (QRDR), although mutations have also been reported in *gyr*B [15]. Genes encoding topoisomerase IV consists of two subunits *par*C and *par*E which have also been shown to be inhibited by fluoroquinolones. In Gram-negative bacteria, topoisomerase IV has been considered as the secondary target to flouroquinolones and alteration in *par*C is involved in the mechanism of resistance [16]. The level of MICs of fluoroquinolones has been shown to correlate with the type and number of amino acid substitution of the target genes within the QRDR [17]–[20]. In our previous study, possible mechanisms of fluoroquinolone resistance were analyzed in clinical strains of *S*. *dysenteriae* type 1 isolated from India, Nepal and Bangladesh [9].

It has also been reported that fluoroquinolone resistance is often the result of a combination of target site mutations and enhanced expression of genes encoding efflux pumps in resistant bacteria [21]. In this study, we report the first isolation of fluoroquinolone resistant *S. flexneri, S. boydii* and *S. sonnei* in Bangladesh. In addition, the mechanism of chromosome mediated fluoroquinolone resistance in *S. flexneri* strains, which results from the combination of target site mutation and energy dependant active pumps involvement has been determined.

## Materials and Methods

### Bacterial strains

A total of 3,789 *Shigella* spp. were isolated from diarrheal patients attending the Dhaka treatment center of the International Centre for Diarrhoeal Disease Research, Bangladesh (icddr,b) between January 2004 and December 2010. These strains were isolated and identified as belonging to different species and serotypes by using standard microbiological, biochemical, and serological methods. Among the *Shigella* spp. 2,181 isolates were confirmed as *S. flexneri,* by using polyvalent commercial antisera (Denka Seiken, Tokyo, Japan). In this study 380 randomly selected ciprofloxacin resistant (Cip^R^) *Shigella* spp. and 25 susceptible *S. flexneri* strains were used. Of these 380, 317 were *S. flexneri*, 3 were *S. dysenteriae*, 4 were *S. boydii* and 56 were *S. sonnei*. For comparison, 12 *S*. *flexneri* 2a strains isolated in Zhending, Hebei province, China in 2002 were included in the study. These isolates were obtained from the collection of the International Vaccine Institute, Seoul, Korea. Of these Chinese strains, 10 were completely resistant and 2 strains showed reduced susceptibility to ciprofloxacin [22]. Details of the representative (n = 25) *S. flexneri* 2a are presented in the Table 1. *Escherichia coli* (ATCC 25922) and *Staphylococcus aureus* (ATCC 25923) were also used as control strains for the antibiotic susceptibility tests.

**Table 1:** MIC and amino acid changes in *gyr*A and *par*C in representative *S*. *flexneri* 2a strains isolated in Bangladesh and China.

Strain	Country	Resistance profile	Plasmidprofile		MIC (mg/L)						Substitutions in QRDR^c^			
				Na	Cip	Cip+CCCP 4mg/L	Nor	Of	Azm	Cro	*gyr*A			*par*C
											Ser-83^a^	Asp-87^a^	His-211^a^	Ser-80^a^
K105	Bangladesh	Amp,Sxt,Na	P1	2	0.008	0.032	0.032	0.047	0.75	0.032	-^b^	–	–	–
K304	Bangladesh	Amp,Sxt	P1	2	0.008	0.032	0.032	0.047	0.75	0.032	-^b^	–	–	–
K6224	Bangladesh	Amp,Sxt,Na,Cip	P1	>256	6	0.5	32	24	0.75	0.064	Leu	Asn	Tyr	Ile
K6806	Bangladesh	Amp,Sxt,Na,Cip	P1	>256	6	0.5	8	16	0.75	0.032	Leu	Asn	Tyr	Ile
K6813	Bangladesh	Amp,Sxt,Na,Cip	P1	>256	8	1	12	16	0.5	0.023	Leu	Asn	Tyr	Ile
K6814	Bangladesh	Amp,Sxt,Na,Cip	P1	>256	6	0.5	16	12	0.5	0.023	Leu	Asn	Tyr	Ile
K10224	Bangladesh	Amp,Sxt,Na,Cip	P1	>256	32	16	16	24	0.25	0.023	Leu	Asn	Tyr	Ile
K6913	Bangladesh	Amp,Sxt,Na,Cip	P1	>256	8	1	12	16	0.5	0.023	Leu	Asn	Tyr	Ile
K9482	Bangladesh	Amp,Sxt,Na,Cip	P4	>256	6	0.5	16	12	0.5	0.023	Leu	Asn	Tyr	Ile
K6915	Bangladesh	Amp,Sxt,Na,Cip	P1	>256	6	0.5	16	24	0.25	0.023	Leu	Asn	Tyr	Ile
K6916	Bangladesh	Amp,Sxt,Na,Cip	P1	>256	8	0.5	12	16	0.5	0.023	Leu	Asn	Tyr	Ile
K9563	Bangladesh	Amp,Sxt,Na,Cip	P4	>256	6	0.5	16	12	0.5	0.023	Leu	Asn	Tyr	Ile
K10347	Bangladesh	Amp,Sxt,Na,Cip	P1	>256	6	0.5	16	24	0.25	0.023	Leu	Asn	Tyr	Ile
CH2535	China	Amp,Sxt,Na,Cip	P3	>256	6	0.5	10	12	1	0.032	Leu	Gly	–	Ile
CH2765	China	Sxt,Na,Cip	P1	>256	6	0.5	16	8	0.5	0.023	Leu	Gly	–	Ile
CH2970	China	Sxt,Na,Cip	P1	>256	6	2	16	12	1	0.023	Leu	Gly	–	Ile
CH2971	China	Sxt,Na,Cip	P2	>256	6	2	24	24	1	0.023	Leu	Gly	–	Ile
CH8603	China	Amp,Sxt,Na,Cip	P2	>256	6	0.5	16	32	1.5	0.047	Leu	Asn	–	Ile
CH9608	China	Amp,Sxt,Na	P3	>256	0.5	0.5	3	2	1.5	0.032	Leu	–	–	Ile
CH9681	China	Amp,Sxt,Na	P1	>256	0.5	0.5	2	2	1	0.032	Leu	–	–	Ile
CH10365	China	Amp,Sxt,Na,Cip	P2	>256	6	0.5	16	16	0.75	0.023	Leu	Gly	–	Ile
CH10416	China	Amp,Sxt,Na,Cip	P2	>256	6	2	24	24	0.75	0.023	Leu	Gly	–	Ile
CH10430	China	Amp,Sxt,Na,Cip	P2	>256	6	2	24	16	1	0.032	Leu	Gly	–	Ile
CH11108	China	Amp,Sxt,Na,Cip	P2	>256	6	2	16	24	1.5	0.047	Leu	Gly	–	Ile
CH12806	China	Amp,Sxt,Na,Cip	P2	>256	6	0.5	16	16	0.75	0.023	Leu	Gly	–	Ile

Abbreviations: Na, nalidixic acid; Cip, ciprofloxacin; Nor, norfloxacin; Of, ofloxacin; Azm, azithromycin; Cro, ceftriaxone, CCCP, carbonyl cyanide-m-chlorophenylhydrazone ^a^Wild type, P1∶140, 4, 2.7, 2.1 MDa; P2∶140, 2.7, 2.1 MDa; P3∶140, 8, 2.7, 2.1MDa; P4∶140, 36–62, 2.7, 2.1MDa.

### Serotyping

Serotype was confirmed using a commercially available antiserum kit (Denka Seiken, Tokyo, Japan) specific for all group factor antigens of *S. dysenteriae, S. boydii* and *S. sonnei* and in case of *S. flexneri* (i) all type and group-factor antigens and (ii) monoclonal antibody reagents specific for all *S. flexneri* type-and group-factor antigens (Reagensia AB, Stockholm, Sweden). Serotyping was performed by the slide agglutination test following a procedure described previously [23].

### Susceptibility testing

Antibiotic susceptibility test was done by disc diffusion method following the guidelines of Clinical and Laboratory Standards Institute (CLSI) using commercially available antibiotic disc (Oxoid, Basingstoke, United Kingdom). The antibiotic discs used in this study were ampicillin (Amp; 10 µg), sulphamethoxazole-trimethoprim (Sxt; 25 µg), mecillinam (Mel; 25 µg), nalidixic acid (Na; 30 µg), ciprofloxacin (Cip; 5 µg), norfloxacin (Nor; 10 µg), ofloxacin (Of; 5 µg), azithromycin (Azm; 15 µg), and ceftriaxone (Cro; 30 µg) [24]. The minimum inhibitory concentrations (MICs) of nalidixic acid, ciprofloxacin, norfloxacin, ofloxacin, azithromycin, and ceftriaxone were determined by the E-test (AB Biodisk, Solna, Sweden).

### Plasmid profile analysis

Plasmid DNA was prepared by the alkaline lysis method of Kado and Liu, with some modifications [25]. The molecular weight of the unknown plasmid DNA was assessed by comparing the mobility of the plasmids of known molecular mass as described previously [26].

### Determination of resistance factor

A conjugation experiment between the multidrug-resistant (Amp^R^ Sxt^R^ Cip^R^) donors, *S. flexneri* serotype 2a K9482and K9563 strains, and the recipient, *E. coli* K-12 (Lac^−^ F^−^), was carried out by a procedure described elsewhere [27]. Transconjugant colonies were selected on MacConkey agar plates containing Amp (50 mg/L). Plasmid analysis and antimicrobial susceptibility testing of the transconjugants were carried out to determine the transfer of plasmids with antibiotic resistance.

### DNA sequence analysis

Chromosomal DNA from selected representative strains was prepared and purified by procedures described previously [28]. Polymerase chain reaction (PCR) for *gyr*A, *gyr*B, *par*C and *pare* were performed according to the procedures described earlier [29]–[32]. PCR amplicons were purified with the GFX™ PCR DNA and gel band purification kit (Amersham Pharmacia, USA). Sequencing of the amplicons was performed using the dideoxy-nucleotide chain termination method with the ABI PRISM® BigDye Terminator Cycle Sequencing Reaction kit (Perkin-Elmer Applied Biosystems, Foster City, California) on an automated sequencer (ABI PRISM™ 310). The chromatogram sequencing files were inspected using Chromas 2.23 (Technelysium, Queensland, Australia), and contigs were prepared using SeqMan II (DNASTAR, Madison, WI). Nucleotide and protein sequence similarity searches were performed using the National Center for Biotechnology Information (NCBI, National Institutes of Health, Bethesda, MD) BLAST (Basic Local Alignment Search Tool) server on GenBank database, release 138.0 [33]. Multiple sequence alignments were developed using CLUSTALX 1.81 [34]. Sequences were manually edited in the GeneDoc version 2.6.002 alignment editor.

### Nucleotide sequences accession number

The nucleotide sequences reported in this paper were submitted in GenBank using the National Center for Biotechnology Information (NCBI, Bethesda, MD) Sequin, version 5.26 under accession numbers- DQ681123-30 for *gyr*A, DQ681105-13 for *gyr*B, DQ681114-22 for *par*C and GQ452292 for *par*E.

### Amplification of *qnr*A, *qnr*B, *qnr*S and *aac(6¢)-Ib-cr*

Multiplex PCR of some representative strains was performed to detect the *qnr*A, *qnr*B, *qnr*S and *aac(6¢)-Ib-cr* according to the procedure described earlier [21]. The primer sequences are 5′-AGAGGATTTCTCACGCCAGGA-3′ and 5′-GGCTGGCCGATTATGATTGGT- 3′for *qnr*A, 5′-GGCTGGCCGATTATGATTGGT-3′ and 5′-CGCGTGCGATGAGATAACC-3′ for *qnr*B, 5′-TGCCACTTTGATGTCGCAGAT-3′ and 5′-CGCACGGAACTCTATACCGTAG-3′ for *qnr*S, and 5′-ATCTCATATCGTCGAGTGGTGG-3′ and 5′-CGCTTTCTCGTAGCATCGGAT-3′ for *aac(6¢)-Ib-cr*.

### Synergy tests

Synergy tests were performed according to the procedure described elsewhere using ciprofloxacin and the efflux pump inhibitor carbonyl cyanide-m-chlorophenylhydrazone (CCCP) [35], [36]. Concentration of CCCP was evaluated and optimized to avoid the cytotoxic effect of CCCP on bacterial growth [37]. CCCP was added to the Mueller–Hinton agar at a concentration of 4 mg/L. Susceptibility testing for ciprofloxacin resistant (Cip^R^) strains was performed by agar dilution plates, with and without CCCP (Sigma Aldrich, USA).

### Pulsed-field gel electrophoresis (PFGE)

Genomic DNA of *S. flexneri* was prepared in agarose blocks and digested with the restriction enzyme *Xba*I (New England Biolabs) according to the PulseNet program descried elsewhere [38]. DNA fragments were separated by pulsed-field gel electrophoresis on a CHEF-MAPPER apparatus (Bio-Rad). Analysis of the TIFF images was carried out by the BioNumerics software (Applied Maths, Belgium) using the dice coefficient and unweighted-pair group method using average linkages to generate dendrogram with 1.0% tolerance values.

## Results and Discussion

**Figure 1:**
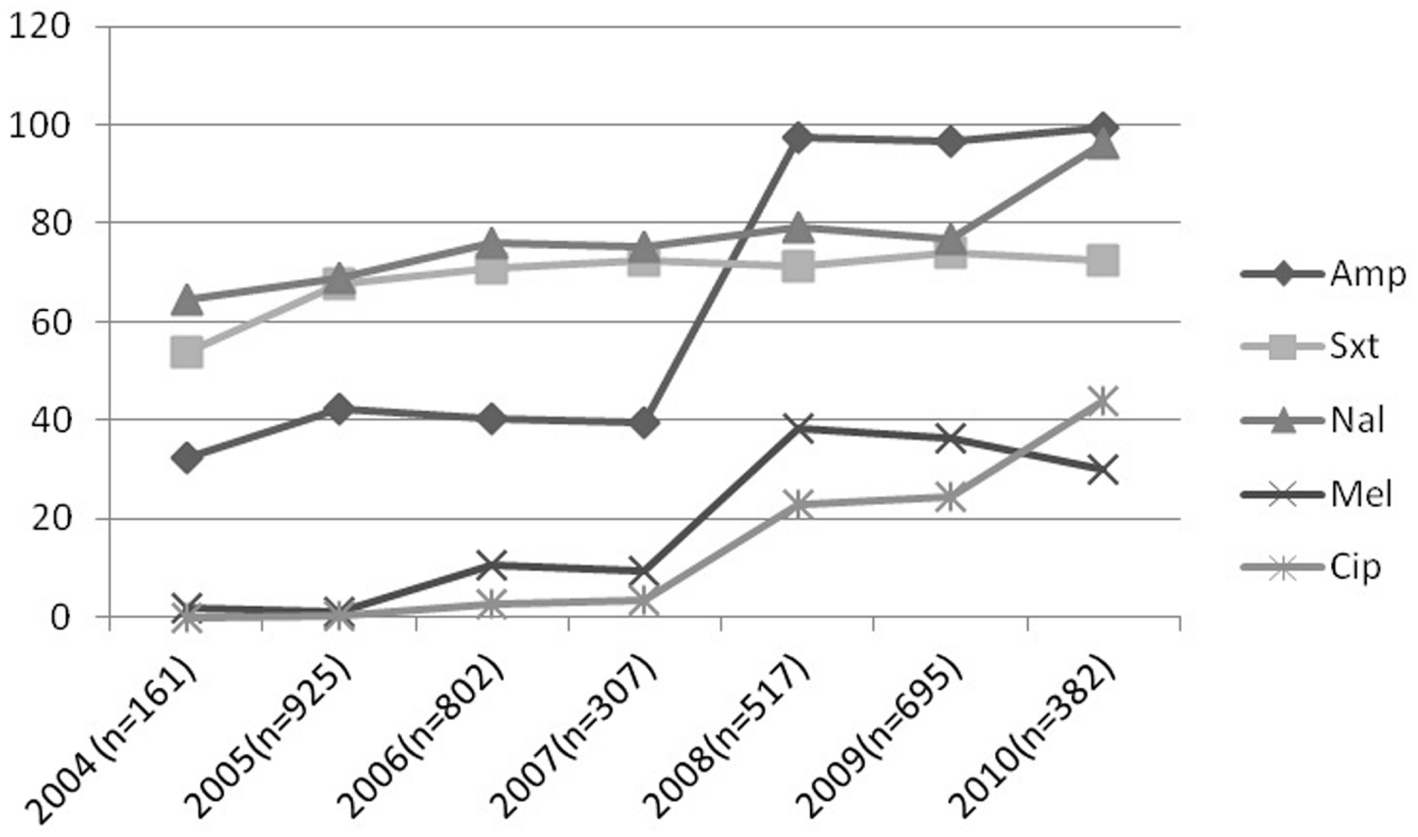
Antibiotic resistance pattern of *Shigella* spp. isolated between 2004 and 2010 in Dhaka, Bangladesh.

**Figure 2:**
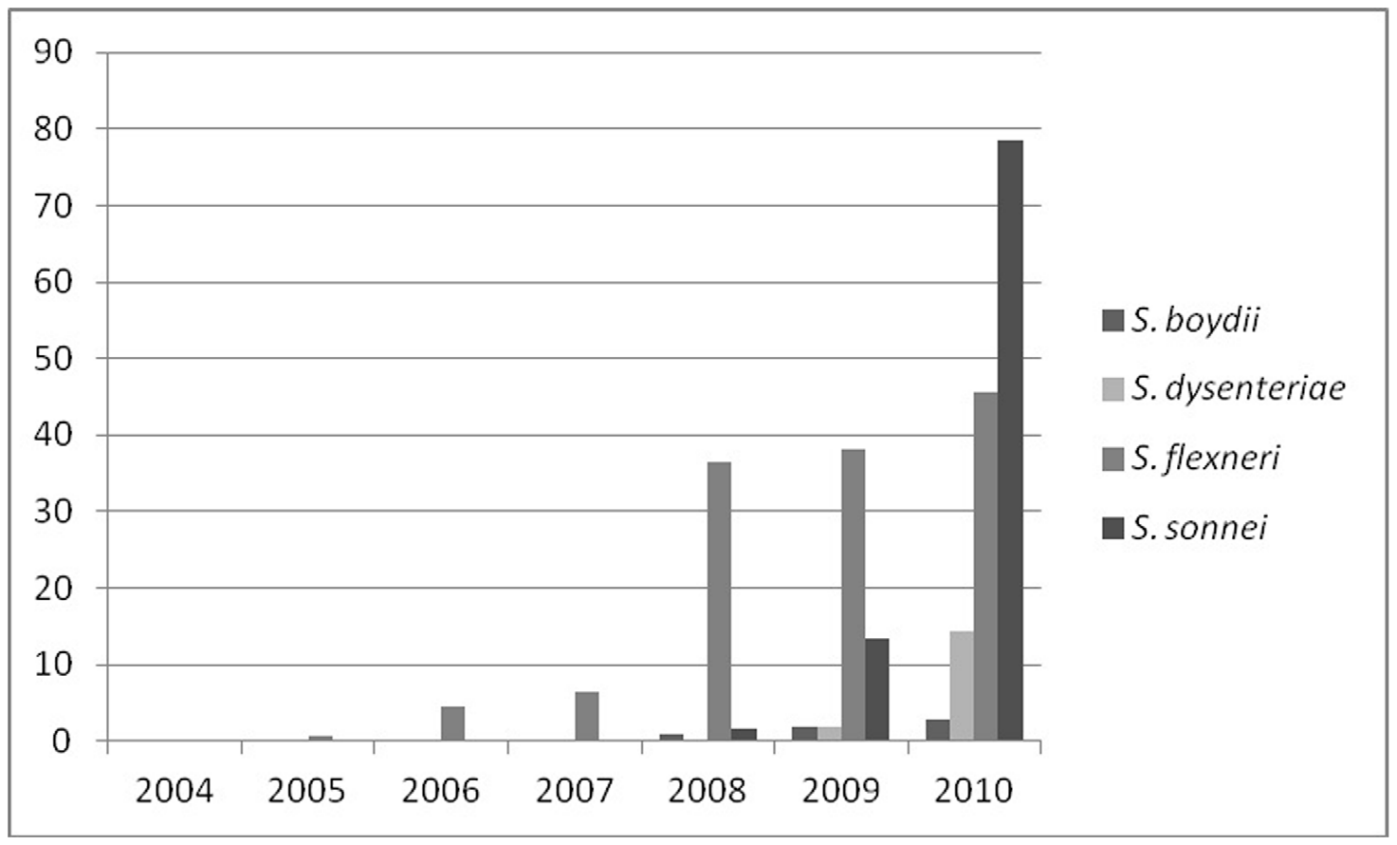
Prevalence of Cip^R^ *Shigella* spp. isolated between 2004 and 2010 in Dhaka, Bangladesh.

Of 3,789 *Shigella* spp. isolated between January 2004 and December 2010 at icddr,b, *S. flexneri* was the predominant serotype (n = 2181, 57.5%), followed by *S. boydii* (n = 753, 20%), *S. sonnei* (n = 574, 15%) and *S. dysenteriae* (n = 281, 7.4%). In *S. flexneri* strains, serotype 2a was the most abundant (40%), followed by serotype 3a (19%), 1b (11%), type 6 (10%), 2b (9%), 1c (5%), type 4 (3%), 4X (2%) and Y variant (1%). Of *S. boydii*, predominant serotype was *S. boydii* 12 (28%), followed by serotype 1 (10%), 4 (9%), 18 (9%), 5 (7%), and 14 (7%). In case of *S. dysenteriae*, predominant serotype was serotype 2 (40%) followed by novel *S. dysenteriae* (17%), serotype 3 (15%), and serotype 4 (13%). Resistance to Amp, Sxt, Na, Mel and Cip in *Shigella* spp. increased from 32%, 54%, 65%, 2% and 0% in 2004 to 99.5%, 73%, 96%, 30% and 44% respectively in 2010 (Figure 1). During the study period, Cip^R^ strains were mostly found among *S. flexneri* (8.3%), followed by *S. sonnei* (1.5%). Among the *S. flexneri* (n = 2181) strains, frequency of resistance to ciprofloxacin (n = 317, 14.5%) increased from 0.7% (n = 3) in 2005 to 45.5% (n = 87) in 2010. Of Cip^R^ *S. flexneri*, serotype 2a was the predominant (96%), followed by type 6 (1.6%), serotype 3a (1.3%), serotype Y variant (0.6%) and serotype 1c (0.3%). Resistance to ciprofloxacin among *S. sonnei*, *S. boydii* and *S. dysenteriae* also started and increased substantially between 2008 and 2010 (Figure 2). It is interesting to note that *S. flexneri* 2a and *S. sonnei* isolated in 2010, 89% (83/93) and 72% (77/107) were resistant to ciprofloxacin respectively.

Ciprofloxacin-resistance was first observed in *S. dysenteriae* type 1 isolated in Bangladesh in 2003 and at the same time isolation rate of *S. dysenteriae* type 1 was abruptly reduced [8]. Since 2004 there is no report of isolation of *S. dysenteriae* type 1 in Bangladesh. Since *S. flexneri* is the most predominant species of *Shigella* and serotype 2a is the most predominant subserotype, *S. flexneri* 2a strains were selected for further detail study in order to understand the ciprofloxacin resistance mechanisms of this pathogen. The range of MIC values of ciprofloxacin, norfloxacin, and ofloxacin for *S. flexneri* 2a, were 6–32 mg/L, 8–32 mg/L, and 8–24 mg/L, respectively (Table 1). These Cip^R^ strains were also resistant to Amp, Sxt, and Na, but were susceptible to Mel, Azm and Cro. The MIC of Cip for 12 strains isolated in China was 6 mg/L and 0.5 mg/L for resistant and reduced susceptible strains, respectively (Table 1).

According to a previous study, at least 4 mutation points at positions 81 (Gly^81^→Ser), 83 (Ser^83^ → Leu), 87 (Asp^87^ → Asn or Gly), and 92 (Met^92^→ Lys) have been identified in the *gyr*A gene associated with quinolone and/or fluoroquinolone resistance of *Shigella* spp. [39]. In the present study, all fluoroquinolone resistant *S*. *flexneri* 2a strains from Bangladesh and China had double mutations in *gyr*A (Ser^83^ → Leu, Asp^87^ → Asn or Gly) and a single mutation in *par*C (Ser^80^ → Ile). A similar type of findings was observed in *S*. *dysenteriae* type 1 strains [8]. In case of Chinese strains, a single mutation at position 83 in *gyr*A (Ser → Leu) and at position 80 of *par*C (Ser→ Ile) was associated with decreased susceptibility to fluoroquinolone but not fully resistance. In addition, difference between Cip^R^ strains isolated in Bangladesh and China was found in amino acid substitution at position 87, which was Asp^87^ → Asn in case of Bangladeshi strains whereas it was Asp^87^ → Gly in case of Chinese strains. However, one of these Chinese strains (CH8603) had the same amino acid substitution at position 87 (Table 1) similar as Bangladeshi strains. This is in accordance with the previous study where double mutations in *gyr*A (Ser^83^ → Leu, Asp^87^ → Gly) were found to be associated with fluoroquinolone resistance in *S. flexneri* 2a strains [40]. It appears that like *E*. *coli*, amino acid substitution in *gyr*A at positions 83 and 87 is common for fluoroquinolone resistance in *Shigella* species. In *E. coli*, mutation in other positions of *gyr*A gene outside the QRDR region of fluoroquinolone resistant strains has been reported, which has yet been reported in *Shigella* spp. [41]. In this study, we had found a new mutation point outside the QRDR region in *gyr*A gene at position 211 (His^211^→Tyr) in fluoroquinolone resistant *S*. *flexneri* 2a strains that was absent in fluoroquinolone susceptible *S*. *flexneri* 2a strains (Table 1). The novel substitution within *gyr*A is however of interest. Further studies such as site directed mutagenesis analysis are suggested to confirm the association between fluoroquinolone resistance and the amino acid substitution at position 211 in *S. flexneri*. No mutations in *gyr*B and *par*E genes were found in any of the *S*. *flexneri* 2a strains in the study, which corroborated with our previous findings [9] and the finding was also compared with the *E*. *coli* control strain (ATCC 25922) to avoid the biasness (data not shown). In addition to substitution in structural genes (*gyr*A/B, *par*C/E), plasmid mediated quinolone resistance has also been reported that can be determined by the presence of the Qnr gene [12]. Several surveys, based on molecular approaches consisting of PCR and sequencing, indicated a higher rate of the association between Qnr-positive and ESBL-positive isolates [42]. In this study none of the strains was found to carry the Qnr gene that excluded the possibility of plasmid mediated quinolone resistance in *S*. *flexneri* isolated in Bangladesh and these result corroborated with our previous observations also [9].

Conjugation experiment was carried out for some selected strains of this serotype 2a and the outcome indicated that resistance to ciprofloxacin is not co transferred with that of ampicillin resistance and not that resistance to ciprofloxacin is plasmid mediated (Table 2). Mechanisms like role of AcrAB-TolC efflux pump and MAR (multiple antimicrobial resistances) phenotype in fluoroquinolone resistance, decreased expression of outer membrane porins, or over-expression of multidrug efflux pumps are also important for detail analysis of fluroquinolone resistance. To elucidate the involvement of efflux pumps in fluoroquinolone resistance in *Shigella* isolates, synergy experiments were performed by using the efflux pump inhibitor CCCP and ciprofloxacin. A degree of synergy between CCCP and ciprofloxacin was observed using the agar dilution method. The concentration of CCCP was evaluated and optimized to control the cytotoxic effect of CCCP on bacterial growth [37]. The MICs of ciprofloxacin resistance were decreased up to 16 fold in an agar plate containing CCCP when compared to those observed in CCCP-free medium (Table 1). These results indicate that efflux pumps may be involved in the fluoroquinolone resistance of *S. flexneri* because there were changes in MICs in the presence or absence of CCCP. It will be interesting to find out the specific mechanisms of efflux pumps mediated fluoroquinolone resistance in *S*. *flexneri*.

**Table 2:** Transfer of resistance plasmid to *E. coli* K-12 by conjugation.

Strain	Parent Strain		Transconjugant	
	Resistance pattern	Plasmid profile (MDa)	Resistance pattern	Plasmid profile (MDa)
K9482	Amp^R^, Sxt^R^, Na^R^, Cip^R^	140, 62, 2.7, 2.1	Amp^R^, Sxt^R^,	62
K9563	Amp^R^, Sxt^R^, Na^R^, Cip^R^	140, 36, 2.7, 2.1	Amp^R^	36

Plasmid profile and PFGE analysis have long been used as molecular fingerprinting tools for bacterial strains. All Cip^R^ strains from Bangladesh were clustered in two plasmid pattern (P1 and P4), while Chinese strains were divided into three patterns (P1, P2 and P3). Plasmids of P2 pattern (140, 2.7 and 2.1 MDa) were commonly present in all strains irrespective of their resistance pattern and origin of isolation.

**Figure 3:**
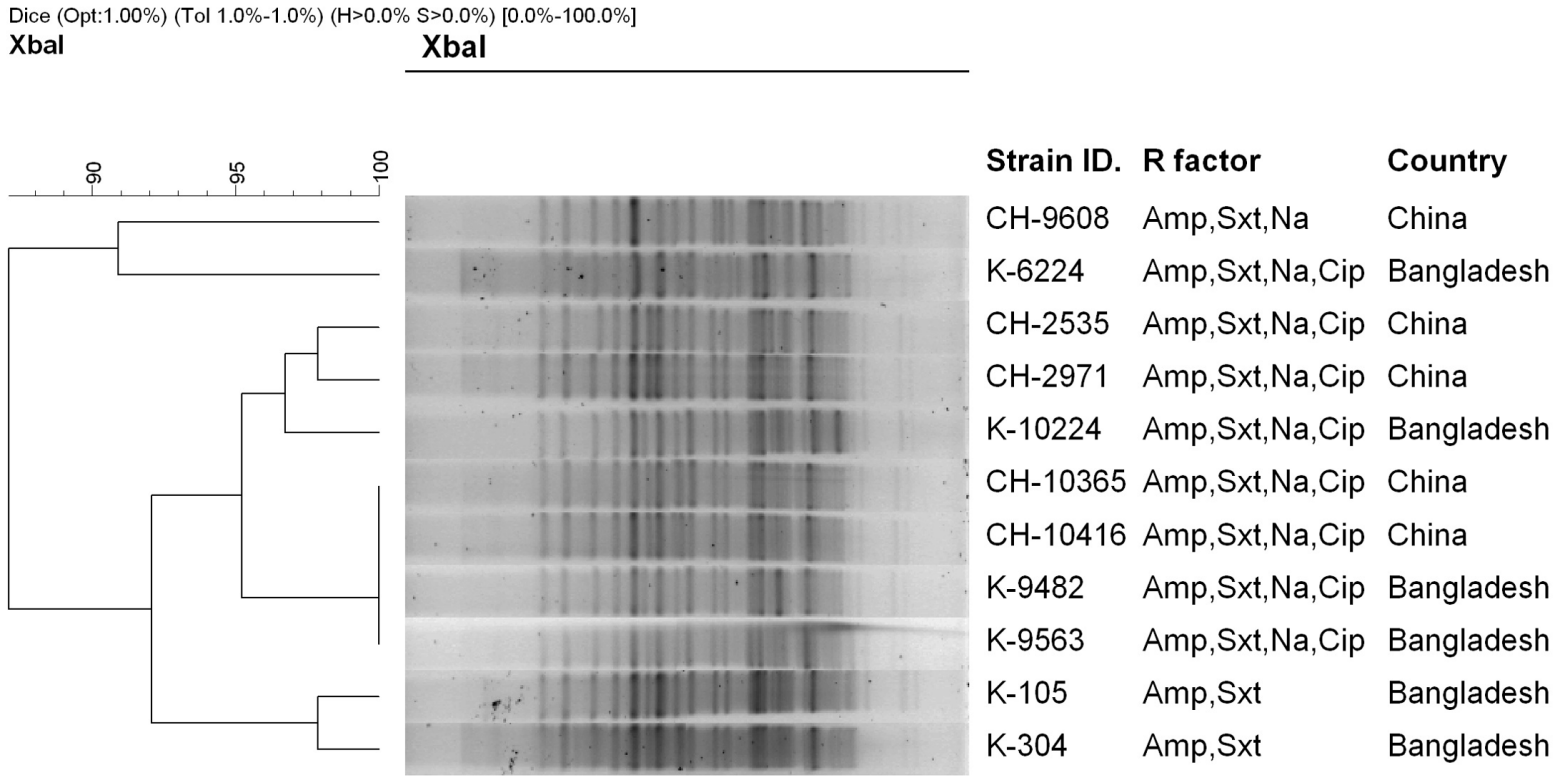
Dendrogram generated by BioNumeric software, showing distances calculated by the dice similarity index of PFGE *Xba*I profiles for *S. flexneri* 2a isolated from Bangladesh and compared with strains isolated in China. The degree of similarity (%) is shown on the scale.

PFGE analysis of representative *S. flexneri* 2a strains isolated from Bangladesh and China, either resistant or susceptible to flouroquinolones revealed a closely related banding pattern with 90% similarity indices. Four *S. flexneri* 2a strains, two from Bangladesh (K9482 and K9563) and 2 from China (CH10365 and CH10416) had identical banding pattern and were member of single clone (Figure. 3) indicating dissemination of a genetically related *S*. *flexneri* 2a in these countries, which is alarming.

Emergence of fluoroquinolone resistance in *S*. *flexneri* 2a has undermined the current treatment strategies of shigellosis as serotype 2a has been identified as the predominant causative agent of endemic shigellosis. Currently, pivmecillinam has been recommended as an alternative drug of fluoroquinolones for the treatment of shigellosis. However, how long can it be used as an effective alternative- is a burning question? In this report, we have shown for the first time the emergence of multi-drug resistance (MDR), especially the fluoroquinolone resistant *S. flexneri* in Bangladesh with their comparative genetic relatedness of the strains from China [43]. This study suggests the need for continuous monitoring of the MDR *Shigella* spp. as well as the elucidation of their molecular mechanisms of drug resistance. This knowledge will help in the development of new antimicrobial strategies, to stop or reduce the emergence and spread of these MDR pathogens.
